# Ag-Based Synergistic Antimicrobial Composites. A Critical Review

**DOI:** 10.3390/nano11071687

**Published:** 2021-06-27

**Authors:** Ekaterina A. Kukushkina, Syed Imdadul Hossain, Maria Chiara Sportelli, Nicoletta Ditaranto, Rosaria Anna Picca, Nicola Cioffi

**Affiliations:** 1 Chemistry Department, University of Bari Aldo Moro, via Orabona 4, 70126 Bari, Italy; ekaterina.kukushkina@uniba.it (E.A.K.); syedimdadul.hossain@uniba.it (S.I.H.); maria.sportelli@uniba.it (M.C.S.); nicoletta.ditaranto@uniba.it (N.D.); rosaria.picca@uniba.it (R.A.P.); 2 CSGI (Center for Colloid and Surface Science), Chemistry Department, University of Bari, via Orabona 4, 70126 Bari, Italy

**Keywords:** silver nanoparticles, hybrid materials, nanocomposites, antimicrobials, synergistic, silver conjugates, chitosan

## Abstract

The emerging problem of the antibiotic resistance development and the consequences that the health, food and other sectors face stimulate researchers to find safe and effective alternative methods to fight antimicrobial resistance (AMR) and biofilm formation. One of the most promising and efficient groups of materials known for robust antimicrobial performance is noble metal nanoparticles. Notably, silver nanoparticles (AgNPs) have been already widely investigated and applied as antimicrobial agents. However, it has been proposed to create synergistic composites, because pathogens can find their way to develop resistance against metal nanophases; therefore, it could be important to strengthen and secure their antipathogen potency. These complex materials are comprised of individual components with intrinsic antimicrobial action against a wide range of pathogens. One part consists of inorganic AgNPs, and the other, of active organic molecules with pronounced germicidal effects: both phases complement each other, and the effect might just be the sum of the individual effects, or it can be reinforced by the simultaneous application. Many organic molecules have been proposed as potential candidates and successfully united with inorganic counterparts: polysaccharides, with chitosan being the most used component; phenols and organic acids; and peptides and other agents of animal and synthetic origin. In this review, we overview the available literature and critically discuss the findings, including the mechanisms of action, efficacy and application of the silver-based synergistic antimicrobial composites. Hence, we provide a structured summary of the current state of the research direction and give an opinion on perspectives on the development of hybrid Ag-based nanoantimicrobials (NAMs).

## 1. Introduction

### 1.1. Antimicrobial Resistance as Unnoticed Threat

The combination of the active functional compounds in order to create a robust composite system is trending within the scientific community: not only are engineering and high-tech research aiming to boost the creation of novel outstanding materials (composite materials for aeronautics and electronics), but the medical field (tissue engineering) and global manufacturers (smart food packaging) also bet on the development of improved, more safe and more functional multicomponent materials [1,2,3,4,5,6,7]. Like in any other craftsmanship, finding the combination of the individual compounds and their properties that will complement each other and work synergistically is a challenging task. Living in the postantibiotics era, providing antimicrobial potential features to the well-known, abundant and safe materials, is one of the key ways to overcome the problems related to the decrease in efficacy of the conventional antimicrobial treatments.

Pathogenic and nonpathogen microorganisms play a key role in the lifecycle of nature and exist in symbiosis with most naturally occurring processes. Microbes are significant in many ways: they are essential for processes like fermentation and microbiota regulation and influence metabolic and hormonal processes of multicellular hosts, including human body regulation of nutrient cycles. Recently, attention has partly shifted from individual microorganisms to biofilms. What is a biofilm, from a general point of view? It is the architectural assemblage of one or multiple types of germs with advantageous collective behavior. It is easily found everywhere in nature. The majority of all microorganisms prefer to live as colonies attached to given surfaces. At this tight arrangement, bacteria exhibit lower growth rates and are protected by surrounding themselves in self-produced extra cellular matrix (ECM) from harsh environmental conditions, making biofilm more resilient to external stress factors like oxidation, radiation, desiccation and so on [8]. ECM has a complex structure. The internal environment is rich with proteins, sugars and nucleic acids and is able to keep bacteria in close vicinity, providing safe and advantageous, tight, cell-to-cell interaction and DNA exchange [9,10]. Recently, it has been found that it is possible to encode light-induced, membrane-potential-based memory-patterns within bacteria in intimate contact as a biofilm [11]. Besides the noxious impact on food, water quality and health care industry, biofilms can serve as a useful and sustainable alternative to provide a low-cost source of power and clean sustainable energy or even act beneficially during cost-effective and sustainable water treatment procedures for some types of filters [12]. Nevertheless, the spread of contagious microorganisms and robust biofilm development is seen as a threat for human health and compromises the quality and performance of various man-made products. Various strategies to inhibit bacterial pathogen growth, reproduction and biofilm formation have been applied, including conventional antibacterial medicines.

Antibiotics became a versatile tool against bacterial infections since the discovery of the very first one, penicillin, and the range of antiviral conventional drugs has broadened over the years and has taken a stable position in the treatment of infections. The question of harm and good impact of modern medicine is more prominent when it comes to the continuous use of antibiotics: microorganisms undergo genetic changes over time in order to resist and survive the application of antimicrobial medicine, acquiring antimicrobial resistance (AMR). There are several factors contributing to the development of resistance that demand constant surveillance in order to understand trends pathogen response to the various changes [13]. Misconceptions on the use of antibiotics, antibiotic resistance and its progression threaten the effectiveness of conventional treatment methodologies on the level of a global concern. Needless to say, the increase in hospital-acquired infections (HAIs) [14,15], foodborne outbreaks [16,17,18,19] and the recent increase in outbreaks of viral infections like Ebola (EVD) in the Democratic Republic of Congo [20] and MERS-CoV in The Kingdom of Saudi Arabia in 2014 [21,22] and worldwide pandemics of H1N1 influenza in 2009 [23] and the most recent SARS-CoV-2 in 2019 [24], which has already become the most commemorative pandemic of the century with highest health, economic and social impact, cause the need to acquire rapid risk assessments and immediate response activities worldwide. Case fatality rate (CFR) in Italy, one of the most stricken countries in Europe by the COVID-19 pandemic, is higher than that of many other countries [25]; one of the possible and logical explanations is that it had been placed on the top of the list of the countries in European Union for antibiotic-resistant deaths [26,27]. Many experts have warned of the direct connection between COVID-19 and AMR [28]. Moreover, AMR is described as “another ongoing pandemic that often goes unnoticed” [29]. WHO had started its global campaign for Containment of Antimicrobial Resistance in 2001, focused on refraining of the unreasonable use of antimicrobials and also encouraging on the development of novel effective treatment agents that do not cause elaboration of AMR. WHO also launched Antibiotic Awareness Day in 2015. Over the past years, there have been numerous statements from leading researchers warning that humanity may face a global pandemic in the near future, a “mass killer”, with potentially catastrophic consequences. Given the increased incidence of infectious outbreaks, there is an urgent need not only on raise of the awareness of the society but also to find effective alternative ways to inhibit pathogen growth, spread and mutations with prolonged positive impact. There are several groups of antimicrobial agents of the post-antibiotic era that were introduced as alternatives to conventional antibiotics and different strategies to fight AMR proposed by the scientific community.

### 1.2. Nanotechnology and Silver

Nanoscale materials represent a large group of versatile tools with amplified and unique properties. Since the rapid rise in numbers of novel technologies for controlled and large-scale production of nanomaterials, nanotechnology became an indispensable part of the majority of industries and fields, vital for humankind. It is expected for nanotech to grow in importance even further; with the progress in theoretical modeling and properties prediction and evolving demand for better performances from areas such as catalysis, electronics and medicine, nanotechnology stands far from oblivion from the scientific community. In particular, metal nanoparticles, widely exploited for use in applications such as diagnostics, providing faster and more accurate medical test results; pharmaceutics, improving distribution of drugs and enhanced effect of therapeutics and treatment with less side-effects; and electronics, allowing the production of smaller devices with superior capacities. These aspects, among many others not listed here, lead to higher implementation and overall increase in performance of materials and improvement in quality of life of people. Notably, silver in a form of nanoparticles, stands out among all nanometals by virtue of its broad intrinsic antimicrobial action [30]. Use of silver in a form of an ion, bulk metal and nanoparticles as antibacterial agents has been known since ancient times [31]. For this reason, it is continually being implemented in diverse branches of the biomedical field. Despite the lack of scientific justification for the effectiveness of silver for antimicrobial treatment during the ancient period and intuitive use for many decades, it was extensively used until the discovery and prompt employment of antibiotics [32]. Emergence of AMR during the antibiotic era and rise of nanoscience made silver and other metal-based nanoantimicrobials (NAMs) objects of close attention once again. Physicochemical properties of nanomaterials follow a specific trend related to size and shape; in particular, for their antimicrobial performance, it has been shown that the smaller the nanoparticle, the higher its capacity to disactivate pathogens [33]. Silver is widely used as a disinfecting agent in health sector facilities and agriculture; AgNPs of different sizes are used in coating compositions for orthopedics and dentistry, as additives in intelligent food packaging materials and as dopants in textile [1,2,34,35]. However, the release, transport and transformation in the relevant conditions, accumulation and long-term effect on health and nature of nanosilver are highly dependent on the size, shape, coating and other factors [36,37]. In spite of all the efforts that have been made, the knowledge gap related to the full understanding of the behavior of nanophases in different forms does not allow us to estimate the total environmental and health impact caused by exposure of AgNPs to the ambient and living organisms [4,5,38].

### 1.3. Compounds of Natural Origin That Exhibit a Wide Range Antimicrobial Action and Do Not Cause Development of AMR

Nature has been an ultimate source of inspiration for technological progress ever since the science was born. Use of natural preservatives to prolong shelf life of food has been known for centuries: antimicrobial herbs, spices, essential oils and many others are applied to improve quality and safety of food systems. Today, taking into account all the safety problems related to the use of conventional antibiotics and metal nanoparticles and working in agreement with the current trend of giving value to sustainable resources, nature also serves as a generous source of natural compounds with inherent growth inhibition ability of pathogenic microorganisms in food [39], biomedical and other important industries. In recent times, extracts and naturally occurring chemicals are widely used in nanoscience: “green” chemistry practice appears as a well-established trend within the scientific community [40,41,42]. Many researchers are focused on the development of novel bioinspired and biocompatible food packaging with multiple functionality: reinforced with antimicrobials, polymer packaging not only protects food products from external environmental irritants, but also prevent food spoilage caused by germs. Besides intrinsic antimicrobial activities, natural antimicrobials have broad physicochemical properties: they express good mechanical stability and good water solubility; these tunable features can bring additional value to the overall performance of the composites [43,44]. Some problems related to direct application are still awaiting investigation: systematic exploration of the structure activity aspects, understanding the mechanism of action and safety trials are under constant study [45]. Nevertheless, there are still a vast number of natural antimicrobials with attractive props matching the need of modern science yet to be explored and applied for the industrial use.

### 1.4. Novel Approaches for the Creation of Organic/Inorganic Composites and Nanofabrication of Synergistic Antimicrobials, Combination with Drugs

**Figure 1:**
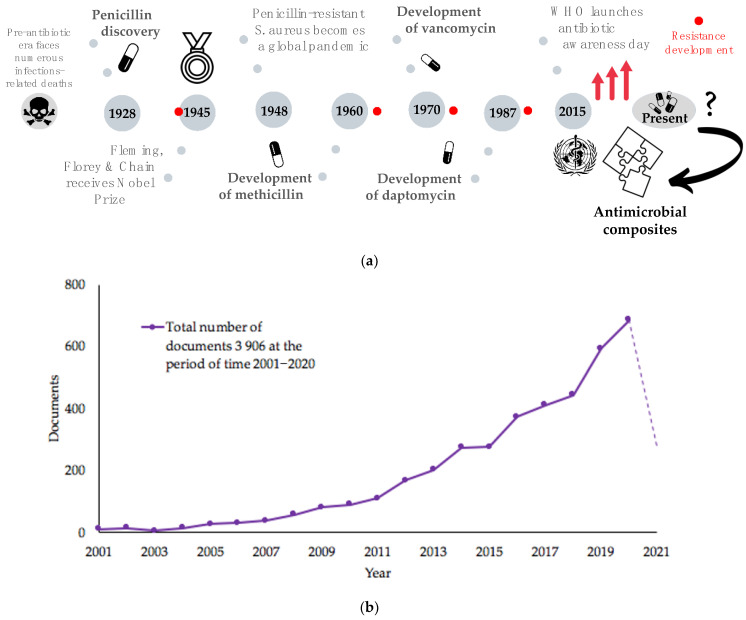
(**a**) Milestones of the antimicrobial medicine and antimicrobial resistance (AMR) development starting from the discovery of penicillin in 1928 and until the present time, where application of antimicrobial composites might work as a missing puzzle in the fight with AMR. (**b**) Statistics on publications in English (articles and book chapters) using keywords “composites” and “antimicrobial”: total number of documents reached 3906 during years of 2001–2020 and is still growing. Scopus. Available online (https://www.scopus.com, accessed on 23 April 2021).

Finding new, efficient, long-lasting antimicrobials that do not cause the development of AMR is becoming more difficult, since microorganisms acquire resistance faster than novel drugs are discovered and approved [46]. Nanotechnology and continuous progress in this field can work as a versatile therapeutic tool to combat antimicrobial resistance, especially against peculiar biofilm features and superbugs. Since resistance mechanisms developed by pathogens toward NPs differ from those associated with traditional antibiotics, a common approach is to apply synergistic strategy against multidrug-resistant hospital isolates: in the presence of AgNPs, the treatment effect is sufficiently higher compared to the antibiotic only [47,48,49]. However, microorganisms rapidly adapt to changes, especially the ESKAPE group pathogens: *Enterococcus faecium*, *Staphylococcus aureus*, *Klebsiella pneumoniae*, *Acinetobacter baumannii*, *Pseudomonas aeruginosa* and *Enterobacter* spp. Thus, novel combination therapies are in high demand, especially ones that do not involve the use of conventional antibiotic drugs alone [50] (Figure 1a,b). Antibiotics in combination, phage therapy, application of antimicrobial peptides and antimicrobial light therapy are the most popularly used alternative strategies [50]. Combination of antibiotics with so-called “resistant breakers” molecules (ARBs) allows an increase in the uptake of the antibiotic through the bacterial membrane and inhibits the defense mechanism of the pathogenic cell [51]. These molecules do not necessarily carry the intrinsic bacteriostatic or bactericidal activity but are being co-administrated or conjugated to failing antibiotics work in synergy to combat AMR. The practical utility of the combination of these methods provides an encouraging outlook to investigate further novel combinations and utilize symbiotic relationships of various compounds. Antimicrobial composite materials might work as robust alternatives to antibiotic-based conjugates, combining several antimicrobial organic or inorganic agents. Silver nanoparticles, whose antimicrobial potency is enlarged due to the nanoscale of materials, can easily undergo in situ and post-synthetic surface modifications with multiple molecules: introduction of contact-killing coatings, stabilization by “green” compounds with naturally occurring antimicrobial potency and complexation with synergistic antibiotics. These strategies allow the functionalization of particles in a way to bring additional properties and enhanced stability, as well as to reduce the usage of AgNPs, minimizing exposure and toxicological effect, a positive point, considering sustainability factors. Mastering the principle of selection of constituent active agents potentiates the efficacy of synergistic nanocomposite.

### 1.5. Aim of the Review

Herein we report the review of inorganic/organic nanoformulations combining silver with compounds that exhibit intrinsic antimicrobial activity and show synergistic performance against various microorganisms, including drug resistant ones. We aim to collect a library of available studies on the enhanced effect of silver-based composites combined with naturally occurring biocompounds with inhibition power toward pathogen growth, biofilm formation and development of AMR. Understanding the mechanisms involved in growth inhibition and consistency of the action of each single composite constituent that leads to pathogen death is the key for creation of novel and safer, long-lasting antimicrobial complex agents. Evaluating the role and the precise effect on boosting the antimicrobial performance by the presence of organic constituents can possibly reduce the content of the inorganic phase and eliminate some questions related to risk assessment of silver nanoparticles. Antimicrobial efficacy of AgNPs in combination with natural antimicrobials such as polysaccharides (chitosan, cellulose and hyaluronic acid derivatives), organic acids (citric, oleic), phenols (tannins, curcumin) and other agents of natural origin (peptides) are critically discussed; toxicological and environmental issues are introduced, as well as mechanisms of antimicrobial action involved behind the synergy. Conclusions on the current state-of-the-art of synergistic nanohybrids are drawn with additional discussion on perspectives in this field.

## 2. General Synthetic Methods of AgNPs

When discussing synthetic methods, generally, division into subclasses grants three main methods of producing silver nanoparticles: chemical, physical and biological [52,53,54]. They allow production of particles of different sizes in forms of nanowires [55,56], nanocubes [57,58], nanospheres [59,60], nanoprisms [61] and many others with more complex architecture [62]. It is worth mentioning that research toward the development of novel synthetic strategies is still ongoing. All the possible variations of precursors, reducing agents and methodologies for AgNPs production are studied and discussed in detail in review papers dedicated to this topic [63,64,65,66]. Therefore, only general criteria, some critical fabrication parameters, pros and cons of each method are briefly discussed in this section. A few examples of Ag-based nanocomposites fabrication are given at the end of this discussion.

### 2.1. Conventional Methods—Chemical Synthesis

Among other strategies, chemical synthesis is the most widely used synthetic strategy for production of metal NPs, including silver. It is cheap and scalable and requires at least two main components for synthesis: a silver salt precursor and inorganic/organic reducing agents, such as borohydrides [NaBH_4_] and sodium citrate [67]. Many review papers have summarized all the diversity of reaction participants, reaction conditions and the features of resulting NPs [30,68]. Control of nucleation and growth process are acquired by the selection of the appropriate starting material and the control of synthetic parameters, such as concentration, reaction medium, pH and temperature. One of the most important steps in the production of monodispersed particles is the stabilization procedure: since the properties of nanoparticles, including antimicrobial activity, highly depend on the size and shape of the particles, prevention of agglomeration and coalescence in a long-term fashion is necessary. The simultaneous reduction and protection of AgNPs can be achieved by the use of agents with dual functionality, for example sugars (fructose, glucose, sucrose) and solid templates like CD-MOFs. Another important step is the purification of the final material from toxic chemicals and side products, which is especially crucial for biomedical application.

### 2.2. Electrochemical Synthesis and Electrospinning Methods

Electrochemical reduction is the one among other techniques which allows one to obtain particles with a high degree of purity and to control particle size by adjusting the reaction time and the current or voltage. It requires simple instrumentation, and synthetic process generally last for relatively short time in comparison to several other routes, e.g., chemical [69]. Different reaction media, the use of steric and electrostatic stabilizers, or even their absence, allow one to prepare particles in a controlled manner [70]. One of the drawbacks of this method for the synthesis of silver NPs is the necessity to use organic solvents and to avoid contamination with water: even the smallest traces of water induce formation of by-products such as silver oxides.

The electrospinning technique is one of the most efficient techniques for production of functionalized polymeric fibers of various diameters and lengths by applying high electric fields [71]. In the past years, many research groups developed composite fibers with inorganic particles, including silver nanoparticles, by this inexpensive process. S.-J. Park et al. have prepared in situ tannic acid stabilized AgNPs within polyurethane nanofibers. This material shows exceptional antimicrobial performance against several common pathogens [72].

In spite of all the advantages these methods offer, there are some drawbacks that limit their abundant usage: the scalability and the need of clean conditions, energy consumption and high sensitivity to the change of the synthetic conditions (humidity, air contamination and others) are the main ones among others.

### 2.3. Physical Methods

In comparison to chemical methods, the physical approach provides an opportunity to use less toxic chemicals without the production of by-products during synthesis [73]. There is a wide choice of physical methods for the fabrication of silver nanophases. Laser ablation in liquids obtains ultrafine NPs in high concentration without agglomeration in a short time and does not require use of toxic chemicals and purification steps. Ultrafine particles with prolonged stability can be obtained, even without use of additional stabilizing agents [74]. Pulsed laser deposition in a vacuum enables preparation of ultrathin films (~3 nm) composed of AgNPs with controlled separation [75]. Moreover, these techniques give the best control over size distribution and are the simplest in comparison to the other techniques [76]. On the other hand, finite scalability, need of advanced equipment and high consumption of the energy limit out of the laboratory application [77].

### 2.4. Bioinspired Methods

Use of natural chemicals for the production of metal NPs becomes as popular and important as traditional approaches due to its eco-friendliness and cost-effectiveness [78]. Lack of toxic and expensive chemicals in the synthetic protocols and use of bacteria, fungi, algae and plant extracts for synthesis lead to highly stable particles with well-defined size and morphology [79]. This low-cost and energy-efficient method is frequently presented as a green alternative route compared to conventional chemical and physical methods and is a subject of an increasing number of publications. One of the finest examples of the green synthesis is the production of metal nanoparticles containing composites by feeding silkworms modified diets: this strategy allows researchers to get functional fibers by impregnating leaves with nanomaterials or their precursors for in situ synthesis [80]. However, all bioinspired methods require longer synthesis time (several hours and even few days) and additional and careful purification step of synthesized material from impurities and by-products [81]. Microbe-mediated synthesis has specific laboratory requirements (sterilized environment and culture conditions), and training of the personnel must be involved [82]. These requirements limit an easy control and scalability of the synthesis of bioinspired approaches.

### 2.5. Development of Complex Composite Systems by Various Synthetic Routes

**Figure 2:**
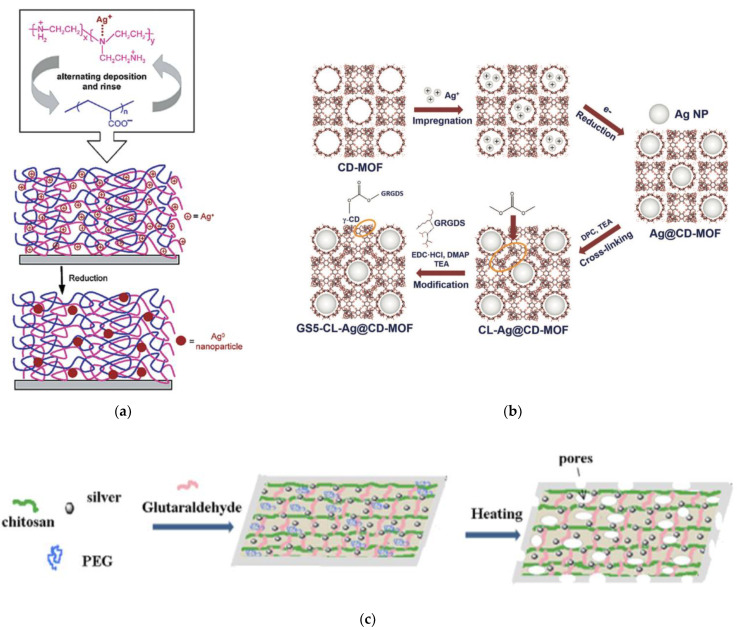

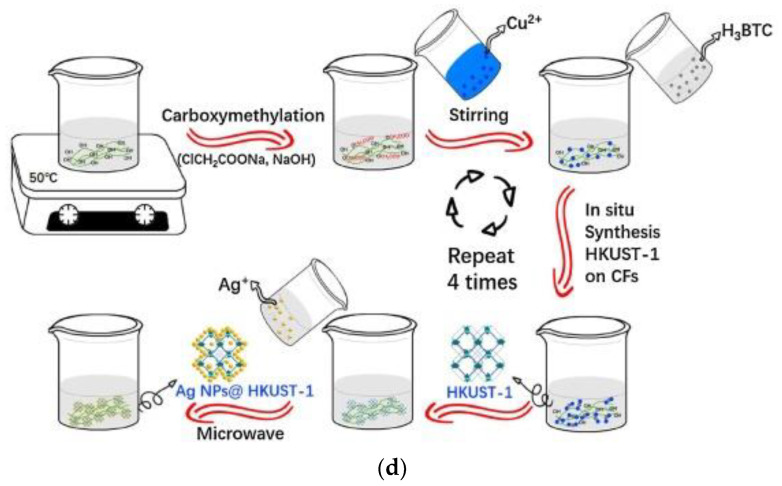
Examples of development of complex composite systems by various synthetic routes. (**a**) PEI–Ag^+^/PAA bilayer. Reduction of silver ions within PEI–Ag^+^/PAA films using freshly prepared NaBH_4_ yielded an abundance of stable nanoparticles within the multilayered system (LBL). Reprinted with permission Nano Letters 2002, 2, 5, 497–501. Copyright 2002 American Chemical Society [83]. (**b**) Schematic diagram of CD-MOF template guided synthesis of AgNPs by solution impregnation, followed by reduction, cross-linking and GRGDS surface modification [84]. Copyright 2019 John Wiley and Sons. (**c**) Schematic illustration of preparation of porous chitosan–silver nanocomposite (PCSSNC) films [87]. Copyright 2010 Elsevier. (**d**) Schematic representation of the in situ preparation of AgNPs@ HKUST-1@ CFs composites [89]. Copyright 2018 Elsevier.

Development of multicomponent nanoantimicrobial systems has drawn attention due to high demand on development of novel high performance hybrid systems. For example, in some LBL systems, AgNPs were reduced in situ by preparing silver–polyethyleneimine (PEI) complexes with further assembly with PPA (Figure 2a). Postdeposition reduction of metal ions yields composite polyelectrolyte films with well-dispersed particles and prolonged release of an antimicrobial agent [83].

Most of the conventional methods lead to poor AgNPs stability and agglomeration. However, use of a solid functional template, which has a dual role of a reductant and stabilizing agent, allows researchers to control size and prevent particle coalescence. One-pot synthesis of ultrafine AgNPs using cavities of porous CD-MOF as template functionalized with antimicrobial GRGDS peptide (Figure 2b) enhances the overall performance of the nanocomposite, including the antibacterial efficacy [84].

Antibacterial nanofiber mats were obtained by electrospinning and cross-linking of PVA/CS/AgNPs blends with glutaraldehyde: chitosan was preliminary used as stabilizer for AgNPs during the reduction process [85]. Incorporation of AgNPs in PVA/CS blends enhanced the electrospinning performance and yielded in the smaller diameter of the e-spun fibers, in comparison to unloaded PVA/CS blends. Antibacterial ability against *E. coli* makes it a promising material for wound dressing application. A similar approach has been reported to fabricate gelatin-based wound dressing material containing AgNPs [86]. Vimala et al. in 2010 reported [87] a multistep fabrication process of porous CS–AgNPs films, which includes as a first step polyol synthesis of AgNPs (by using PEG and CS), and production of glutaraldehyde-crosslinked chitosan films (Figure 2c). The developed composites were found to be active against classical test strains *E. coli*, *Bacillus* and *K. pneumoniae*, bacterium, which is notorious for causing respiratory nosocomial infections in hospitals [88]. Another example of template use is the immobilization of AgNPs via in situ microwave-assisted reduction on the surface of highly porous HKUST-1/CFs composite with inherent antibacterial activity. It resulted in a biocomposite with superior inhibitory activity against *S. aureus* [89]. HKUST-1, MOF made up of copper nodes with 1,3,5-benzenetricarboxylic acid struts between them, served during the reduction of silver ions as stabilizer with high surface area and improved stability and dispersity of AgNPs (Figure 2d).

## 3. Mechanisms of Antimicrobial Action

For a better understanding of the synergistic behavior of composites, the interaction of neat components with microorganisms, in particular with bacteria, should be mentioned. Several reports give a clear view on the possible mechanisms that lie behind bacteriostatic and bactericidal action of inorganic [30,90,91,92,93,94], organic [95,96,97] and hybrid [98,99] nanophases. There are several factors contributing to the inhibition effectiveness of nanophases, and scale matching (scale factor) of all the participants (microorganism and NAM agent) is among the most important ones. The strong antimicrobial action of AgNPs is associated with their nanoscale size and unique properties. The exact mechanism(s) of action is not yet fully understood, remaining a challenging problem under debates to solve. Several possible ways of interaction between AgNPs and pathogens have been introduced over the years.

**Figure 3:**
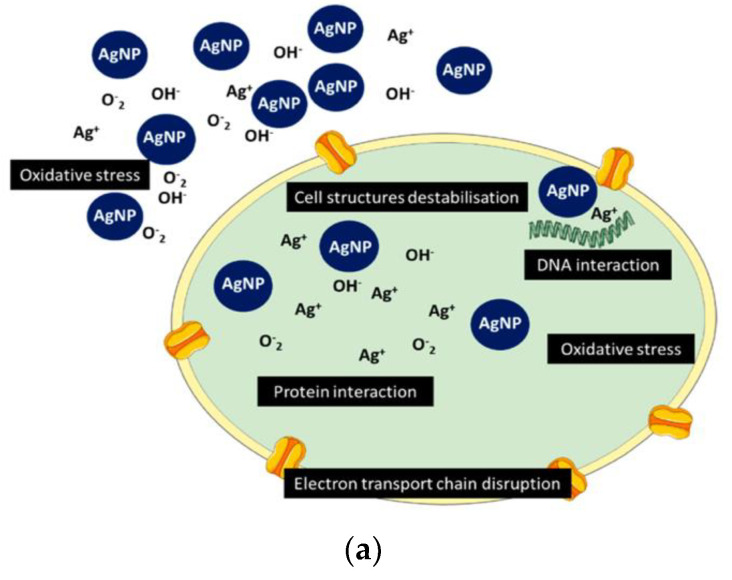

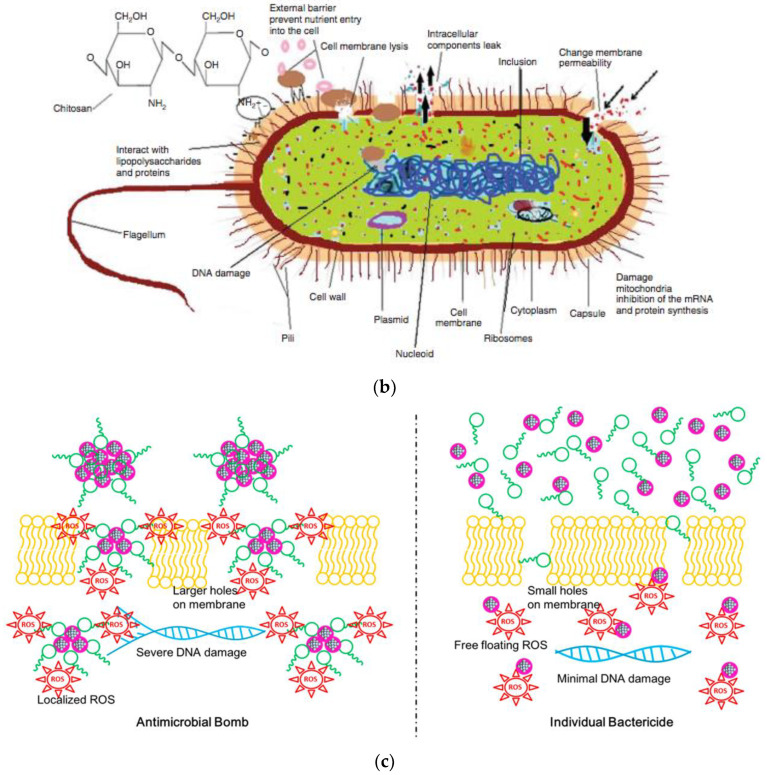
Overview of the mechanisms of interaction of antimicrobials with the pathogens. (**a**) Schematic representation of AgNP mechanism of antimicrobial activity [30]. (**b**) Various antimicrobial mechanisms of chitosan [103]. (**c**) The schematic representation of the mechanisms of action and their differences of individual bactericides, silver nanoclusters (AgNCs) and antibiotic daptomycin (D) and of the antimicrobial hybrid bomb (D–AgNCs) Reprinted with permission ACS Nano 2016, 10, 7934–7942. Copyright 2016 American Chemical Society [107].

For AgNPs, two main killing pathways are known: interaction with microbial membrane with further disruption of permeability and functioning of membrane (e.g., via interaction with thiol groups of cysteine) and penetration and action inside the bacterial cell [100]. Production of ROS in close proximity with bacteria and inside of them causes oxidative stress; interaction of silver species with DNA and proteins disrupts the life cycle and leads to cell apoptosis (Figure 3a) [30,101].

Depending on the structure of the organic compound, the mechanism of interaction differs. For positively charged chitosan molecules, interaction with bacteria is due to electrostatic forces: microbial cell membranes carry negative charge on their outer surfaces. This model of action is the most acceptable: electrostatic interaction between antimicrobial and the membrane results in simultaneous interference. First, by changing the properties of membrane wall permeability, there is an appearance of the internal osmotic imbalances, which leads to inhibition of the growth of pathogens. The second interference is caused by the leakage of the intracellular electrolytes (e.g., potassium ions, proteins, glucose) due to the hydrolysis of the peptidoglycans in the pathogen wall [102]. Additionally proposed mechanism are: chelation of metals, binding of chitosan moieties with microbial DNA and inhibition of the genetic replication, suppression of spore elements and binding to essential nutrients to microbial growth (Figure 3b) [103]. In a similar way, CS suppresses the replication of the viruses: it initiates defense responses that affects and inhibits replication of RNA [104].

Several possibilities have been proposed with regards to the synergy of the nanohybrids: the synergistic effect of composites could be a combination of different mechanisms of antimicrobial action of the unadulterated components or consequence of collaborative and coordinated action of all the constituents of the nanohybrid. Potara et al. give two possibilities for synergistic effect of CS/AgNPs composite: first, that covering the nanoparticle surface with chitosan leads to steric stabilization and inhibits or cancels aggregation potential, thus increasing the effective concentration of particles capable of interacting with the cellular surface; the other possibility is that the positive charge coming from the chitosan biopolymer to the particles enhances their binding to negative charges present at the cell surface [105,106].

Another peculiar example of the antimicrobial hybrid mechanism of action is presented by the combination of the silver nanoclusters (AgNCs) with the well-known antibiotic dantomycin (D) [107]. These bactericides have high efficiency against bacterium, but when conjugated together, D + AgNCs hybrid has an effect of an antimicrobial bomb. The sufficient damage of the bacterial membranes and enhanced and continuous local production of ROS within the pathogen structure leads to severe DNA damage and prevents resistance development, which is demonstrated in Figure 3c. Zheng and his team showed that physically mixed D and AgNCs was less efficient in bacterial killing.

Nevertheless, careful investigation of the exact mechanism of interaction is needed in each specific case when it comes to the organic/inorganic composite.

## 4. Silver-Based Composites with Synergistic Antimicrobial Activity: Organic Parts of the Composite with Intrinsic AM Action

### 4.1. Polysaccharides and Derivatives

Chitosan (CS) is derivative of a natural widespread polymer chitin, which is the second most abundant polysaccharide after cellulose [108]. Annual production of chitin from marine species for various industrial fields reaches several millions of tons and is considered to be a cheap source of production of commercially important polymer chitosan [109,110]. Its nontoxic, biocompatible, biodegradable, anti-inflammatory and antimicrobial, unique biological properties allow application of CS and CS-based biomaterials in the most important branches of medicine and food-related industries [111,112]. Due to its cationic nature, CS has been shown to have various biological activities, most importantly an extensive and pronounced antimicrobial mode of action against a wide range of pathogens [113]. It is well-known that CS has an intrinsic property to inhibit and kill pathogens [114]. So as potential application, it can be used not only as AgNP-biocompatible delivery system but as an amplifier of the antimicrobial Ag-based agents against pathogenic microorganisms. Indeed, the chitosan–silver composite’s (CS/AgNPs) dual mechanism of action has received consideration from scientific community in the last two decades. While other reviews are mostly centered on the methods of synthesis [115,116,117], CS and CS derivatives-based composites containing AgNPs and their properties are reported in this paragraph, with focus and extended discussion on some selected works and study of the combined effect of both organic and inorganic antimicrobials acting together against bacteria, viruses and fungi.

The very first paper dedicated to preparation and characterization of a nanocomposite consisting of both chitosan (CS) and commercially manufactured silver nanoparticles (AgNPs) was reported in 2006 by Rhim and co-workers [118] as potential intelligent food packaging material. In this study, neat chitosan films and CS-based films with incorporated antimicrobial agents were tested against several microorganisms. As classical bacteria used to test antimicrobial power, CFU values of Gm+ bacteria *S. aureus* and *L. monocytogenes* and Gm− *S. typhimurium* and *E. coli* were significantly lower for both abovementioned films in comparison to control samples, which indicates intrinsic antimicrobial functions of CS films and synergistic action of CS/AgNPs.

To the knowledge of the authors of this review, in 2008, the first paper was published by Sanpui and group [119] in which CS was used simultaneously as a reducing and stabilizing agent to produce environmentally friendly and stable nanocomposites for application in catalysis. One-pot synthesized particles were uniform in size distribution, with a mean diameter determined by TEM of ~5 nm and stable, thus resulting in high surface-to-volume ratio of nanoparticles and high catalytic efficiency. In the next study, the same group successfully determined the antibacterial activity of CS/AgNPs composite against recombinant *E. coli*.

**Figure 4:**
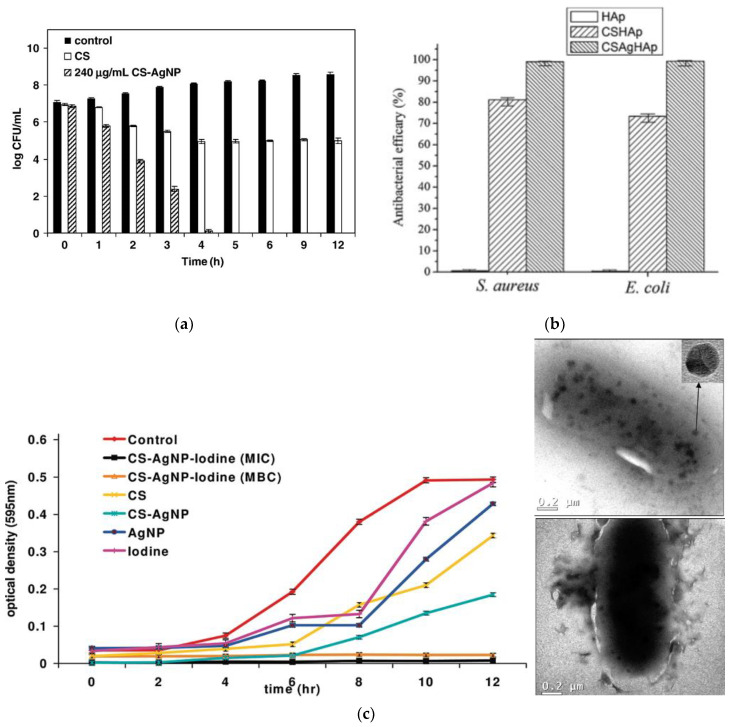
Examples of the result of the combination of polysaccharides and derivatives with AgNPs. (**a**) Comparative effect of CS–AgNPs composite and chitosan, alone (with same 0.024% conc. of CS in both the samples), on recombinant *E. coli* viability. CS represents chitosan [120]. Copyright 2008 Elsevier. (**b**) Antibacterial effect of the prepared CSAgHAp coating against *E. coli* and *S. aureus* strains [143]. Copyright 2015 Elsevier. (**c**) Effect of different concentrations of iodinated chitosan–AgNP (at MIC and MBC) and individual components of the composite on the growth of GFP recombinant *E. coli*. Sample TEM micrographs showing interactions between silver nanoparticles in the iodinated composite and bacteria. Reprinted with permission from Langmuir 2010, 26(8), 5901–5908. Copyright 2010 American Chemical Society [156].

It is shown in Figure 4a that the composite was able to fully inactivate bacteria within 4 h, while polysaccharide, alone, worked only for the growth inhibition of *E. coli* [120]. Later in 2008, Wei and co-workers [121] explained CS/AgNPs interaction by carrying out FT-IR measurements. The drastic decrease in the NH stretching and the blue shift of the NH bending band suggest successful attachment of silver nanophase to nitrogen atoms of amino groups; the appearance of the new band on the spectra, which might be attributed to carbonyl stretching, indicates oxidation of hydroxyl groups in the polymer molecule during the in situ reduction of Ag^+^ to Ag^0^. The authors performed extended antibacterial tests against *E. coli*, along with detailed bactericidal kinetics tests for strains of *E. coli*, *S. aureus* and nonpathogenic *B. subtilis*. For all three strains, it was proven that the developed antibacterial composite has a dual mechanism of action: it not only successfully inhibits bacterial growth by cationic effect of CS but also kills bacteria by bactericidal action of AgNPs.

Seeing that fast and long-lived antibacterial performance is possible to achieve by immersion of AgNPs in the cationic polysaccharide matrix, other teams were encouraged to apply the same strategy for the synthesis of similar systems to test their ability to inactivate a variety of pathogenic microorganisms. The effect of particle size, reaction conditions (temperature, presence of oxygen, solvent), metal–polymer interaction and ionic release are crucial for the evaluation of collective antimicrobial activity of polymer–metal composites.

For example, Kumar-Krishnan and team [122] in 2015 compared antibacterial properties and other properties of two types of bionanocomposites, CS/AgNP and CS/Ag^+^ ion. In spite of the successful performance of CS/Ag^+^ ion against (Gm+) and (Gm−) bacteria, AgNPs stabilized by CS showed superior activity toward inhibition of *S. aureus* and *E. coli*. The synergistic effect of metal NPs and CS resulted in two orders of magnitude higher bactericidal effect for the same concentration of silver.

Wang et al., among the first groups in 2015, reported [123] one-step production of uniform-sized CS spheres of ~2 mm with AgNPs immobilized on the surface with average diameter of 15 nm for antifungal tests. The authors found in their study that CS spheres, alone, have active antifungal behavior against *C. militaris*, an abundant mushroom that is frequent in various fields [124], and *A. cinnamomea*, which causes destruction of the inner cavity of aromatic tree *Cinnamomum kanehirai* [125]. Consequently, Ag@CS composite gives a higher rate of synergistic selective inhibition of growth against both tested fungi.

Within the next few years after the first report about successful merge of AgNPs with CS for antimicrobial purposes, studies dedicated to the development of similar two-component systems with different reaction conditions and a variety of resulting sizes of AgNPs appeared available in the literature. Moreover, the first review dedicated to antimicrobial CS/AgNPs nanocomposites was publishes in 2015 [126]. The facile method of in situ synthesis in acetic water solution by both chemical [127] and Y-radiation [1,128] resulted in long-lived CS-stabilized AgNPs with sizes less than 6 nm, and antibacterial tests observed a decrease in colonies for *E. coli* and *Bacillus*. Swelling capacity of polymer matrix has a great importance on overall antimicrobial performance, as it was shown in several studies [87,129]. Indeed, it had been found that biocomposites with higher porosity exhibit greater effects as antibacterial agents due to the enhanced release of AgNPs. Although discussion on this aspect is still open, one of the possible explanations for amplification of bactericidal action could be caused not only by AgNPs but also by cationic effect of the available CS matrix environment [130]. In 2013, Mori and team [131] further exploited antimicrobial action of CS/AgNPs nanocomposites against H1N1 influenza A virus. Particles of different sizes, 3.5 nm, 6.5 nm and 12 nm, were embedded into CS matrix in aqueous medium and tested for antiviral performance. The tendency between antiviral activity and size of the particles was found to be the same as for antibacterial cases: more pronounced activity was observed for composites containing smaller AgNPs, having higher active surface area for interaction with outer membrane of the virions. The authors claim that neat CS does not expose antiviral activity against A/H1N1; on the other hand, studies present in the literature indicate that CS and CS derivatives could be potentially used as antiviral agents against Influenza A [132], Newcastle virus [133] and Tobacco Mosaic virus (TMV) [134]. Unfortunately, the application of metal-based CS composites as potential agent for antiviral therapy is not as widely investigated as naked AgNPs [135,136,137].

In spite of many attractive properties, chitosan has poor mechanical performance and bad stability in the presence of the aqueous media. Hence, the application of nonreinforced chitosan-based composites in the form of films, sponges and so on is limited, especially in biomedical field where biological media provide constant exposure to aqueous bioenvironment. Hydroxyapatite (HA), the major inorganic feature of the bone, and its synthetic analogue are widely used as an osteoconductive coating for metallic artificial body parts [138,139]. Application of ceramics for orthopedics implants face the problem of implant rejection due to internal inflammatory processes on the surface of the prosthesis [140]. Thus, in order to overcome this disadvantage, numerous reports involve fabrication of water-insoluble and biocompatible, hydroxyapatite-supported composites, including both CS and AgNPs, as a source of antimicrobial power [141,142,143,144]. The first paper using this approach was reported by Pang and Zhitomirsky [145], in which researchers used electrophoretic deposition in a mono- and multilayered fashion on various conductive substrates (e.g., stainless steel). They were the first ones to demonstrate the possibility to combine the functional properties of different materials, like mechanical robustness of HA and release of antimicrobial Ag^+^. However, the authors did not report tests of antibacterial activity and did not provide discussion on the role of CS. In 2011, Saravanan and colleagues [146] reported preparation and characterization, including antibacterial activity measures, of CS/AgNPs/HA scaffolds for biomedical application. The role of chitosan and its involvement in antimicrobial performance in the composite is explained by means of zone inhibition tests against *E. coli* and *S. aureus*: for both types of scaffolds, with and without AgNPs, this activity is observed, evidently with higher values for silver-containing systems. Another study support the theory of synergistic activity of CS/AgNPs composites. In 2015, Yan et al. [143] demonstrated by the plate counting method that HA coating, alone, does not show any antibacterial efficacy, whereas values of activity against *E. coli* and *S. aureus* corresponding to the composite coating were found to be 81.2% and 73.4% for CS/HA and for CS/AgNPs/HA 99.1% and 99.3%, respectively (Figure 3b). Low solubility of chitosan in basic and neutral media limits its biocidal ability demonstration, as chitosan is active only in acidic conditions [147]. To further exploit synergistic activity of CS/AgNPs composites, some groups applied the functionalization of the chitosan to enhance its performance as antimicrobial agent. One of the most popular potential approaches is biocomposite formulation for wound dressing and tissue engineering with variable composition and increased complexity of the nanocomposites [148,149,150]. For example, one of the most cited works dedicated to the development of chitosan-based wound dressings was reported by Ong and co-workers in 2008 [151]. The authors used a combination of CS, AgNPs and polyphosphate polymer (PP) as procoagulant with intrinsic antibacterial ability [152]. When dissolved in water, polyphosphate forms an anion, leading to a formation of polyelectrolyte complexes with cationic chitosan. CS/PP was found to be bactericidal against both *P. aeruginosa* and *S. aureus*, though with a slower kill rate and reduced time effectiveness compared to CS/PP/AgNPs. Another interesting example of application for wound dressings is provided by Travan et al., in which lactose-modified chitosan, Chitlac, was used [153]. In particular, its acidic solution was used for the reduction of silver ions to metallic form and further mixed with an alginate, thus generating a three-dimensional highly hydrated hydrogel. Chitlac/AgNPs composite showed tremendous bactericidal effect against *S. epidermidis*, *E. coli*, *S. aureus* and *P. aeruginosa*. Explicit killing kinetics tests against *S. epidermidis*, known for the ability to form biofilms that grow on catheters and other surgical devices and act as a common cause for hospital-acquired infections [154], displayed very fast capacity of this formulation to inactivate bacteria cells after 2 h of treatment. Another similar study with Chitlac was reported in 2013 by Marsich and team [141]. AgNPs were prepared in the presence of Chitlac and followed by absorption on the alginate/HA scaffold. Even though the activity of the AgNPs with other stabilizer (e.g., without intrinsic antimicrobial activity) was not compared to Chitlac/AgNPs, the results indicated that impregnation of scaffolds resulted in almost full inhibition of the growth rate of bacteria.

Another exciting approach was introduced by Banerjee et al. [155], where the combination of three antimicrobial agents was used: CS matrix, AgNPs and molecular iodine, which is well-known disinfectant and antiseptic [156]. The antibacterial properties of the individual components as well as iodinated composites were tested against recombinant GFP-expressing *E. coli*, and MIC and BIC values were determined. Iodinated CS/AgNPs nanohybrid exhibited higher activity at much lower doses of the individual components (Figure 4c). Positively charged CS, known to intimately interact with negatively charged cell walls, and the presence of AgNPs in the proximity to the cell created pores on the bacterial cell surface, thus increasing interaction effectiveness (TEM Figure 4).

The AgNPs-containing CS acetate dressing effectiveness was tested on infected burns on mice [157]. D. Huang et al. found that the survival rate was higher in the case of AgNPs/CS acetate-treated groups than for nonsilver composites and the untreated group, thus showing significant synergistic effect against Gm− *P. aeruginosa*. Ishihara and co-workers have demonstrated the great potential of silver-impregnated, chitin-based composites and nanofiber sheets, CNFS/AgNPs, as a disinfectant for wound dressings, clothes, plastics and PPE (masks). Activity of different types of chitin combined with AgNPs was evaluated: the stronger the interaction of organic host due to increased available binding areas for AgNPs, the higher bactericidal performance of the nanohybrid was demonstrated. Homogeneous dispersion and tight absorption of AgNPs on CNFS resulted in higher antimicrobial activity against *E. coli* and influenza A virus than those of AgNPs, alone [158]. When CS was combined with cellulose to form composite films, substantial inhibition zone was observed against *E. coli* (~1.2 cm) and *S. aureus* (~0.8 cm); upon conjugation of AgNPs to the films, antibacterial activity has been improved even further, with values ~2.6 cm and ~2.0 cm, respectively [159].

Not all polysaccharides have inherent bacteriostatic power like CS; thus, current research aims to innovate modified natural polysaccharides with robust antimicrobial activities and other additional functionalities. Polysaccharides like cellulose and hyaluronic acid derivatives demonstrated ability to amplify antimicrobial performance when combined with AgNPs. Chemically unmodified cellulose does not exhibit intrinsic antibacterial properties; some cellulose derivatives, on the contrary, enhance antimicrobial performance of the individual antibacterial agents. For instance, when conjugated with cellulose, agents like AgNPs have more pronounced activity against several pathogens [160,161]. Functionalization with the antibacterial group, e.g., via covalent attachment of the quaternary ammonium salt or amine groups, can endow a cellulose-based membrane robust antibacterial properties [162]. Oxidized cellulose can react with antimicrobial molecules containing amino groups: cotton fabric treated with triazole with and without AgNPs was tested for inhibitory performance against *E. coli* and *S. aureus*, and all the samples showed high bacterial reduction percentage, even after multiple washing cycles, in comparison to untreated fabric [163]. Hyaluronic acid (HY) and its derivatives exhibit strong intrinsic antibacterial and antiadhesive properties [164]. HY-functionalized nanomaterials have been found useful in drug delivery, molecular imaging and as diagnostic agents in cancer therapy [165]. HY doped with various metal NPs, e.g., Au, Ag, Cu and AgPd, offers safety and capacity needed for its use in the preparation of medic dressings [166]. Addition of AgNPs to the composition of HA-based nanohybrids enhances antimicrobial performance: the HY/AgNPs nanocomposite wound dressing fabric has higher antibacterial effect against Gm− *E. coli* and stronger mechanical properties compared to plain HY fabrics [167]. Moreover, the addition of metal NPs leads to higher wound healing efficacy of this nanohybrid.

### 4.2. Phenolic Compounds

Polyphenols is another attractive large category of natural organic compounds with known native antioxidant properties and low toxicity [168,169]. They are found in vegetables, fruits, legumes and cereals [170] and are also produced by some microorganisms [171]. The broad variety of plants containing polyphenols has attracted attention due to availability and low cost for industrial use, including chemistry-related fields. Many polyphenolic compounds are known to exhibit not only antioxidant activity but also significant bactericidal and antifungal activity [172]. For example, polyphenol-rich tea infusions [173], rosemary extract [174] and wine tannins [175] demonstrated strong abilities to inhibit prominent Gm− and Gm+ bacteria. Individual components like Curcumin (diferuloylmethane), caffeic acid and gallic acid have shown a strong antibacterial activity against various bacteria and fungi [176,177].

For many years, bactericidal and anti-inflammatory activity of Curcumin (CCM), an important constituent of common spice turmeric or *Curcuma longa*, has been often reported and widely exploited [178,179]. Its activity has been found to be pronounced against common human pathogens, Gm+ *S. aureus* and *E. faecalis* and Gm− *E. coli* and *P. aeruginosa*. For the last example of bacteria, Rudrappa et al. reported that CCM inhibits biofilm formation [180]. To further exploit the antibacterial activity, CCM was used simultaneously with common antibiotics to test synergistic activity against resistant strains [181]. Mun and co-workers used combination of the antibiotics oxacillin, ampicillin, ciprofloxacin and norfloxacin with CCM to reduce MIC values of Methicillin-resistant *S. aureus* (MRSA) [182,183]. In addition, inhibition of biofilm formation has been reported by several groups [184,185].

Synergy to fight pathogens and inhibit their activity could be achieved not only through a combination of natural biocide CCM with antibiotics and other conventional agents but also with metal NPs, which exhibit pronounced antimicrobial activity. Numerous works report use of CCM as a reducing and stabilizing agent for synthesis of AgNPs for antibacterial and other applications [186,187,188,189]. Despite the fact that most of the studies focus solely on the antibacterial activity of synthesized metallic NPs, there are evidences present in the literature that shine light on the fact that use of curcumin as complementary antimicrobial agent brings additional antibacterial contribution [190].

Varaprasad and colleagues used CCM as an additional antimicrobial component to impregnate hydrogel containing AgNPs for antibacterial and wound dressing applications [190]. Although CCM was not involved in the synthesis and capping process of metal NPs, binding CCM to hydrogel materials brought additional inhibition activity against test bacteria *E. coli*: AgNPs/hydrogel/CCM composites have demonstrated a 90% reduction rate of bacterial growth compared to AgNPs/hydrogel and hydrogel/CCM systems with an efficiency of less than 25%. Release of CCM and AgNPs from the hydrogel network was sustained, and the correlation between concentration of AgNPs within the network and CCM loading was proposed: the more AgNPs available for conjugation with CCM, the higher overall loading of organic phase will be.

**Figure 5:**
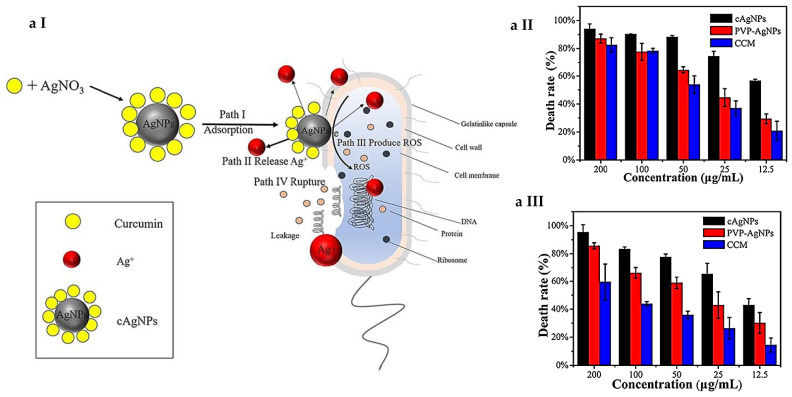

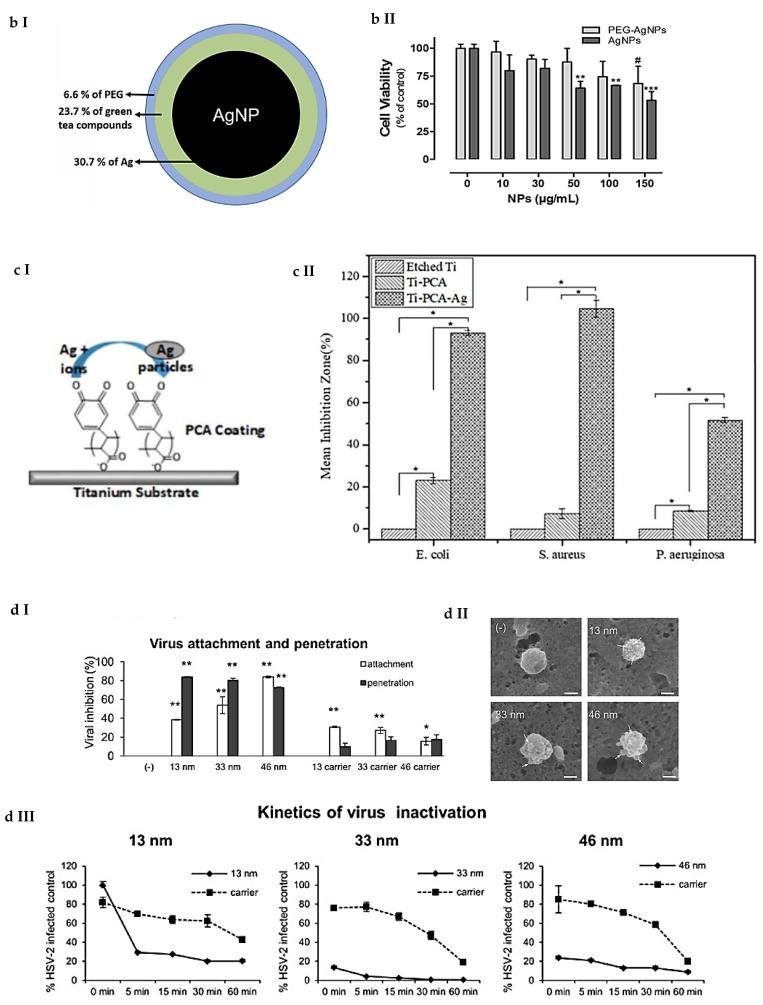
Examples of the result of the combination of phenolic compounds with AgNPs. (**a**) (**I**) Schematic diagram illustrating the fabrication and bactericidal process of cAgNPs as a kind of synergistic antibacterial agents; cell death rate of (**II**) *B. subtilis* and (**III**) *E. coli* bacteria after incubation with cAgNPs, PVP–AgNPs and CCM at different concentrations (12.5, 25, 50, 100, 200 μg/mL) [191]. Copyright 2019 Elsevier. (**b**) (**I**) Schematic representation of the obtained AgNPs capped with green tea extract compounds and PEG: (**II**) HaCat cell viability after incubation with AgNPs and PEG-AgNPs at different concentrations for 24 h [198]. Copyright 2019 Elsevier. (**c**) (**I**) Schematics of the process of synthesis of metallic silver (Ag) on the polycaffeic acid (PCA) on etched titanium (Ti) substrate Ti–PCA [200]; (**II**) graph of inhibition zone for three strains of *E. coli*, *S. aureus* and *P. aeruginosa* for PCA, PCA–Ti and PCA–Ti–Ag [201]. (**d**) (**I**) Viral inhibition (%) for virus attachment and penetration experiments with the use of 33 nm and 46 nm AgNPs and 13 nm AgNPs and corresponding carriers; (**II**) SEM images in EDS mode of HSV-2 virus incubated with AgNPs of different sizes (13, 33, 46 nm).White arrows point out on the interaction spot of the NPs with the surface of virion; white bars indicate 100 nm; (**III**) kinetics of AgNPs and HSV-2 interaction expressed as % of HSV-2-infected positive controls [203].

Song et al. [191] studied in detail the mechanism of CCM/AgNPs composite interaction with the bacteria and synergistic antibacterial activity (Figure 5a I). As a control PVP-stabilized AgNPs (PVP/AgNPs) composite and naked CCM were chosen to quantify and compare MIC and FIC values. Both PVP/AgNPs and CCM showed minor inhibition activity against proliferation of *E. coli* and *B. subtilis*, with MIC values for PVP/AgNPs at 32 μg/mL and CCM at 64 μg/mL, whereas the CCM/AgNPs composite was highly synergistic, and the MIC value was reduced to 8 μg/mL. Survival rate of cells was quantified and showed that CCM/AgNPs were able to disrupt the membrane structure of bacteria, leading to the successful killing of pathogen (Figure 5a II and III).

The use of plant extracts, rich with polyphenols and other natural reducing and capping agents, for the synthesis of metal NPs has drawn significant attention due to the availability of the natural starting materials, simple and eco-friendly synthetic protocol and possibility of bringing new virtues to the capped nanoparticles. Tea extracts, which are known for antioxidant activity [192] and antitumor effects [193], widely applied in green synthesis of potentially biocompatible AgNPs [194,195,196]. Antibacterial and antiviral activities of polyphenolic tea compounds have been studied and exploited for food safety and animal and human health applications [197]. In 2019, Rolim and co-workers developed a one-pot method to synthesize AgNPs using green tea extract (TE) [198]; they further covered NPs with PEG, so the tea compound stayed on the surface of the NP but under the layer of the polymer, as it shown in Figure 5b II. MIC and MBC values have demonstrated inhibitory dominance for TE/AgNPs over PEG/AgNPs. It was suggested that the presence of the phytochemicals might contribute to enhanced antibacterial performance against *E. coli*, *P. aeruginosa* and *S. enterica*: they might disturb the key processes related to the bacterial cytoplasmatic membrane [199] and allow AgNPs to better and faster enter and disrupt the pathogen cell. Withal, the concentration of TE/AgNPs required to achieve pronounced antibacterial effect was not toxic to human skin HaCaT cells (Figure 5b II). Another interesting example is provided by Lee et al. [200], with polycaffeic acid (PCA) and AgNPs used in synergistic antibacterial combination as a coating of titanium surface for application in orthopedics (Figure 5c I). Although the main role of PCA is to, in situ, reduce and immobilize AgNPs by binding to quinone and semiquinone moieties of PCA, it was found that, even with the presence of single PCA coating, antibacterial effect can be acquired. The main antibacterial property is due to the Ag^+^ release and demonstrated by ICP-OES, but the ability of polyphenolic compound to increase membrane permeability also contributes to the overall effect, which is supported by graph of inhibition zone in Figure 5c II [201]. Matei et al. in 2018 provided a detailed study on the activity of four polyphenol inclusion compounds, CCM, ferulic acid, gallic acid and silymarin, in the complex composite with and without AgNPs against *Fusarium culmorum*, a fungus that causes diseases of cereals worldwide [202]. All of them possessed superior antifungal activity against this pathogen in two different dispersion media (chitosan hydroalcoholic solution and deep eutectic solvent), especially composites containing ferulic acid and CCM; it was demonstrated that the addition of AgNPs improved the efficacy of the composites. The decrease in effective concentrations EC50 (reduces mycelial growth by 50%) and EC90 (reduces mycelial growth by 90%) values down to 62% and 51%, respectively, in the case of CCM/AgNPs-based composite, suggests synergy in antifungal performance.

Due to increased need on the development of novel antiviral agents, Ag-based nanocomposites have a potency to act as versatile inhibitors of viral pathogens. Orlowski and co-workers provided an example of the antiviral effect of tannic acid-modified AgNPs (Ta/AgNPs) against Herpes Simplex Virus Type 2 (HSV-2) [203]. TA/AgNPs of different sizes (13 nm, 33 nm, 46 nm) were able to inhibit the viral attachment to the host cell surface (Figure 5d I); the process of inactivation is faster and with higher efficiency (approx. 80%). The efficiency has the highest rate when the TA/AgNPs and HSV-2 virions are in a direct interaction with each other, which was confirmed by SEM (Figure 5d II). Tannic acid potentially binds to the glycoprotein spikes of the virion and prevents attachment and the entry of the virus to the host cell, hence the rapid replication; biological affinity and shape- and size-dependent interaction of AgNPs with the virion result in the deactivation of the replication of the pathogen (Figure 5d III). The spike distance and AgNPs’ size-dependent manner of interaction may play crucial role in the kinetics of virus inactivation process.

Both AgNPs and phenolic compound exhibit intrinsic ability to suppress the growth and kill various pathogens, and, in combination, they give an amplified effect. Without a doubt, more detailed studies on the consistent mechanism of pathogen cell interaction with complex phenol-based antimicrobial agents are required in order to evaluate the sequence and force of interaction of distinct components.

### 4.3. Organic Acids

Use of organic acids as both reducing and capping agent is in accordance with green chemistry principles and works as a safer alternative to conventional sodium borohydride, which may cause environmental and biological damages. Combinations of different organic acids are also used for better control over yielding material parameters such as size and shape during the synthetic process.

Oleic acid, an example of a fatty acid with long alkyl chain and head carboxyl group, has been found to be a versatile tool to control size and shape of AgNPs during the reduction process [204,205,206]. Antimicrobial lipids exhibit direct and indirect inhibitory effects against various pathogens [207]. There are several mechanisms of antibacterial activity involved in the process of killing of the pathogen. Oleic acid and other fatty acids destabilize bacterial cell membranes by direct interaction with membrane enzymes and other membrane-associated proteins. Increased cell permeability and cell lysis and disruption of the electron transport chain interrupt determinative vital processes. These activities cause inhibition of cell growth or cell death. It has been proposed that oleic acid has bactericidal effect against a broad spectrum of Gm+ and Gm− bacteria, including group *A streptococci*, gram-negative pathogens responsible for a number of skin-associated suppurative infections [208,209]. The antibacterial activity against *E. coli* and *S. aureus* of oleic acid-stabilized AgNPs was reported by Le et al. in 2010 [205,210]. Oleic acid-stabilized particles with average size ~10 nm showed four times higher inhibition activity toward the tested pathogens in comparison with myristic acid-capped AgNPs, indicating the additional contribution is due to the presence of profound bacteriostatic effect from an organic constituent with intrinsic antimicrobial activity.

Citric acid is a well-known natural preservative used as an additive in food industry and plays an important role in a life cycle of many living organisms, being the key element of the Krebs cycle [211]. Being recognized by FDA as safe direct food additive (E330) and having no accepted daily intake (ADI) limit, it is widely used for the acidification of nutrition products or packaging of food. It displays activity against common food pathogens such as *E. coli*, *S. aureus*, *S. typhimurium*, *L. monocytogenes* and many others [212,213,214]; biofilm formation of some pathogens (*E. coli* and *Salmonella* sp.) is noticeably inhibited, owing to the anti-quorum-sensing potential of the acids [215]. Depending on the synthetic route, citrate ions can play the role of a reductant, a complexant and a stabilizer in the formation of AgNPs, and citrate-coated particles have been reported to be less toxic in comparison with other types of coatings [216,217]. Potential application of citrate-coated AgNPs includes sequestering heavy metals in aquatic environment to lower toxicity and bioaccumulation [218]. Their fate after exposure to the environment and interaction with external matter has also being investigated due to the variety of application possibilities [219], e.g., inhibiting mycotoxin production [220] and resistant breast cancer cells protein function [221]. Taking into account broad activity range, some groups reported comparison of citrate-coated particles with AgNPs stabilized with other capping agents. There was no clear trend on the effect of the coating on antibacterial efficacy, only trends proving metal particle size-dependent antibacterial activity of AgNPs-based hybrids. It has been proposed that solubility might be a more important determinant of antibacterial activity than the surface charge, as such is case of citrate-coated AgNPs, as well [222]. It was possible to increase inhibition performance of cellulose nanofibrils (CNFs) films treated with commercial solution of AgNPs against Gm+ *L. monocytogenes* and Gm− *S. enteriditis*; the antimicrobial effect was potentiated by the addition of 5% citric acid in the CNFs [223].

Intrinsic antimicrobial activity of gallic acid (GA) against pathogens such as *E. coli*, *P. aeruginosa*, *S. aureus* and *L. monocytogenes* was reported [224]; Sorrentino and group demonstrated that GA is potentially applicable as a biocontrol tool to preserve fresh black truffles from spoilage: this acid not only inhibits growth of storage-associated bacterium *Pesudomonas* spp. but also exerts strong antimicrobial action against other undesirable microorganisms such as *Eumycetes* [225]. Being rich with phenolic groups, GA is used for the synthesis of metal nanoparticles as a reductant and stabilizer for application in catalysis [226], sensing [227] and enhancing cancer radiotherapy efficacy [228]. Combined reinforced antimicrobial effect of GA-coated AgNPs has been shown by Matei et al. against *P. cinnamomi*, one of the most pernicious pathogens in agriculture [229,230]: the highest fungicidal activity corresponded to GA–AgNPs in comparison with the use of bare AgNPs and other coated composites.

Overall, the use of safe natural organic compounds as fraction of a composite can address additional questions of food and drug safety interventions. Mechanistically, the disruption and permeabilization of the outer membrane of bacteria by natural antimicrobials enhances the bactericidal effect of AgNPs: the surface chemistry might play a defining role in the microbicidal action of complex nanostructures despite their size and shape-dependent properties. By providing strong negative surface charge by coverage with citrate shell, Ag-based composites show increases in activity toward Gm+ bacteria; consequently, the surface, covered by positively charged species, has higher binding affinity with Gm− germs.

### 4.4. Peptides

Antimicrobial peptides (AMPs), short positively charged chains (2–50) of linked amino acids, is a diverse class of naturally abundant components. It is well-known as a base for the fabrication of theranostic agents [231] and viable alternative to current therapeutic strategies and one of the most promising and safe tools to bypass the resistance mechanisms expressed by pathogens [232]. Besides antibacterial activities, AMPs possess the broad spectrum of activity, including anti-bacteria, anti-fungi, antiviruses, and anticancer [233]. Several studies are dedicated to study of diverse mechanism of antimicrobial action of various peptides [234,235] against pathogenic microorganisms and interaction with biofilm components [236,237]: host defense peptides (HDP) easily bind to negatively charged bacterial surface and cause membrane malfunction, enabling it to inhibit and disperse biofilms and cause very low levels of induced resistance [238]. Synthetic and semisynthetic, nature-inspired peptides show both biofilm inhibition and eradication activity against a broad range of pathogens, including resistant ones: many review papers summarize all the recent advances and application of AMPs [239,240,241,242,243,244,245,246,247]. Just to provide a few examples, bioconjugation of small AMP to PET has been found to be an effective strategy to fabricate “active” food packaging with intrinsic conservation function [248]; application of HA-binding peptides in dentistry has been reported to be a promising agent for inhibition of dental plaque growth [249]; polydopamine peptide coating of catheters shows excellent ability to inhibit catheter-associated urinary tract infection-relevant microbes (CAUITI) [250]. Various combinations of cationic AMPs with other peptides and conventional antibiotics allow broadening of bacteriostatic and bactericidal performance of traditional antimicrobials and simultaneously lower the possibility of resistance genesis. Reduced cytotoxicity of inorganic phase and enhanced performance against both Gm+ and Gm− germs make these composite potential candidates in practical application, like wound healing and wound care [251,252,253]. Engineering of AgNPs-based composites with AMPs has been reported by several groups within the last decades. In 2009, Ruden studied synergism between the number of AMPs and silver-based materials: silver nitrate and 25 nm sized AgNPs [254]. The following conclusions have been made: there is pronounced synergy of antimicrobial effect by combining peptides with AgNPs; generally, nanophases show higher amplification effect than silver (I) ions against most of the strains; peptide–AgNPs conjunction does not increase hemolysis—thus, this property indicates possible therapeutic window.

**Figure 6:**
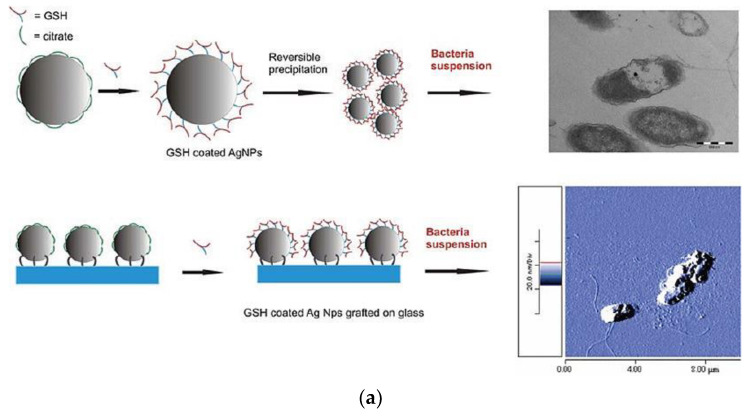

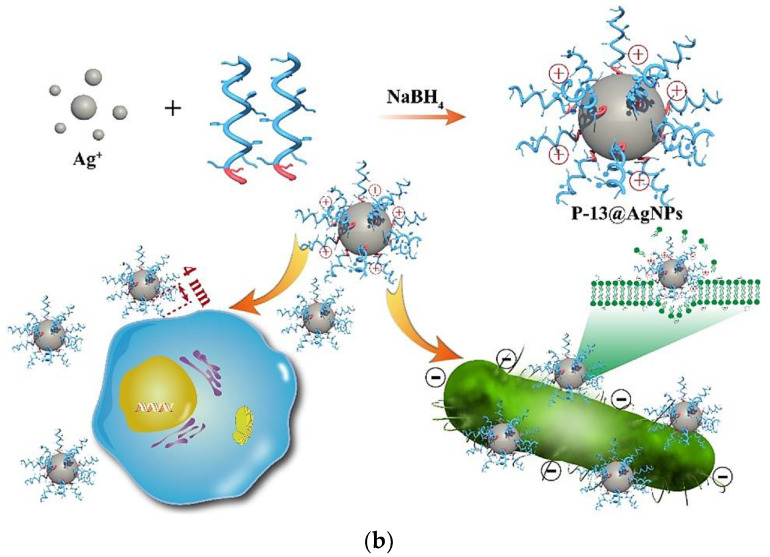
Examples of the result of the combination of peptides with AgNPs. (**a**) Schematic representation on GSH–AgNPs composite grafted on glass with TEM and AFM images of *E. coli* treated with GSH-coated NPs [255]. Reprinted with permission from Langmuir 2012, 28, 8140–8148. Copyright 2012 American Chemical Society. (**b**) Proposed mechanisms for reduced cytotoxicity and enhanced antimicrobial activity of P-13@AgNPs. Silver ions are reduced to AgNPs in presence of P-13 peptides using NaBH_4_ as a reductant. The existing of P-13 between AgNPs and cells isolates the toxic Ag^+^ from cells in the distance (theoretically ~4 nm, with a stretched peptide standing on the surface of AgNPs), which dramatically reduces the cytotoxicity effect on cells. In contrast, the P-13 peptides also endow highly positive charges on the surface of AgNPs, benefiting from the attracting interactions between P-13@AgNPs and negatively charged bacteria [257] Copyright 2020 Elsevier.

The selection of suitable peptides is the important preparation step before the actual development of Ag-based composites, since not all the peptides bring enhanced antimicrobial activity upon combination with metal nanophases. Taglietti et al. demonstrated the reduced effect of GSH-coated AgNPs toward inhibition of Gm+ *S. aureus* with a thicker outer peptidoglycan layer: naked AgNPs grafted on a glass substrate had higher microbicidal activity, whereas “long-distance” mechanical action of grafted particles was limited when GSH was present as a coating (Figure 6a) [255]. A few mechanisms should be taken into account: short-distance nanomechanical action of AgNPs base and direct ion release upon penetration of nanocomposites inside the cell, which is less pronounced in the case of Gm+ bacteria. Liu et al. tested a novel AgNPs–peptide (AgNPs–pep) composite against *E. coli*, *B. subtilis* and *C. albicans* and compared antimicrobial activity to a number of other silver-based materials: silver ions, CA-coated AgNPs, triangular SDS–AgNPs, and core-shell silver–gold system [256]. However, notwithstanding the smaller effective diameter in comparison with CA-coated AgNPs, the microbicidal activity was higher for AgNPs–pep. G3R6TAT peptide has a strong influence on cell penetration effect, and once Ag–pep translocates into the cell, reinforced growth inhibition takes place, in the case of *B. subtilis*. On the contrary, the less negatively charged surface of *E. coli* slows down the affinity and exhibits higher penetration resistance of positively charged Ag–pep. Interesting insights on the peptide–nanoparticle core-shell structure were reported by Gao et al. to explain reduced toxicity of the NAMs. Independent-designed peptide P-13 during single step reaction created a protective shell around the AgNPs (Figure 6b). The shell thickness could be up to 4 nm when the peptides were in a stretched states; thus, they inhibit the cytotoxic activity of silver [257].

Bioconjugation of AMPs and NPs could reportedly be used for the treatment against number of pathogens [258], including mycobacteria [259]. Bioconjugation of AgNPs with nisin showed superior antimicrobial activity against food spoilage microorganisms, which was higher than for a peptide and nanometal, alone [260]. The possibility to perform peptide-mediated green synthesis of AgNPs and fabrication of various nanocomposite structures, e.g., nanofibers [261], is emerging as a budding combinative approach, one of the most promising among others [262]. It should be mentioned that various synthetic methods allow conjugating of peptides to metal nanoparticles without structural changes of the organic part [263].

## 5. Overview on Pros and Cons of Ag-Based NAMs Application

There are advantages and disadvantages related to the use of Ag-based hybrid NAMs, which are listed in Table 1. The organic component provides additional amplification of the antimicrobial properties thanks to the difference from nanometal mechanism of interaction with pathogens. In some cases, for example with chitosan, phenolic compounds and organic acids, the advantage consists of the fact that they serve as stabilizers and reducing agents. Thus, the synthetic steps are limited, with shorter time needed for the development of the final products. The main disadvantage related to all the hybrids is the lack of studies related to their simultaneous mechanism of action, toxicity and destiny in the environment. However, these drawbacks have a great potential to be overcome in the near future, since more and more research groups are focusing on the detailed and extensive study of these hybrids NAMs.

## 6. Concluding Remarks and Perspectives

Silver-based hybrid nanomaterials represent a wide range of novel materials with still-to-be exploited properties and applications. Numerous opportunities for combining conventional antimicrobial agents of natural or synthetic origin with AgNPs open the way to create new alternatives to fight AMR. By tuning the composition of multicomponent systems, it is possible to trigger different mechanisms of pathogen destruction and prevent resistance development. By the provided examples in the reviewed literature, we saw that the simultaneous use of inorganic and organic phases reinforces the overall performance in inhibiting and destructing pathogens and biofilms. Different mechanisms of biocidal and biostatic actions have been proposed; thus, to gain deeper understanding on the exact roles of single components, additional microbiological studies are required. Overall, the positive aspect on the creation of robust multicomponent NAMs is that the time consumption and financial investments for creation of antimicrobial composites are much lower than needed in the case of the development and approval of the new antibiotics.

There is a great perspective for antimicrobial hybrid materials development and efficacy investigations from the practical point of view: cosmetic, food, pharmaceutical and many other industries require fast, cheap and scalable technologies for the implementation of the highly efficient materials with intrinsic bactericidal and bacteriostatic properties with reduced toxicity to fight pathogenic germs that develop resistance against conventional antimicrobials.

## Figures and Tables

**Table 1:** Summary table with organic antimicrobials and the features of their use in combination with nanosilver.

Class of Organic Compounds	Organic Antimicrobial Compound	Advantages on Combination with AgNPs	Particular Features of the Compound
Polysaccharides and derivatives	Chitosan	Can be conjugated with AgNPs in many ways, both complex and one-pot synthesis (can be used as both stabilizing and reducing agent); system thoroughly studied, including toxicity and stability	Easily available, cheap, does not need additional modifications
	Chitosan derivatives	Provides flexibility in terms of complexation/conjugation with AgNPs and structural organization of the composite material; antimicrobial activity could be reached in a controlled manner	Tunable and enhanced antimicrobial activity; can be designed against specific pathogens
	Cellulose derivatives	Allows functionalization with a wide range of antibacterial groups; provides better mechanical stability and broadens the potential application	
	Hyaluronic acid and derivatives		Provide strong antiadhesive properties; one of the main compounds used in tissue engineering
Phenolic compounds	Curcumin	Can be conjugated with AgNPs in many ways, both complex and one-pot synthesis (can be used as both stabilizing and c agent);	Pronounced activity against common human pathogens; abundant and biosafe
	Tea extracts	Provides reduced or no toxicity to human cells	Have antibacterial and antifungal activity; tannic acid possess antiviral activity
	Acids (polycaffeic, tannic, ferulic)	Increased membrane permeability to allow silver ions and ROS attack pathogens more efficiently	
Organic acids	Oleic acid	By providing strong negative surface charge, they show increased activity toward Gm+ bacteria	Provide several mechanisms of antibacterial activity; highly applicable in food safety
	Gallic acid		
	Citric acid		
Peptides	Host defense peptides (HDP)	Provide possible therapeutic window	Can be designed against specific pathogens
	Independent-designed peptides		

## Abbreviations

ADI	Acceptance Daily Intake
Ag–pep	Silver-Peptide Conjugate
Ag/AgNPs/NPs	Silver/Silver Nanoparticles
AgNCs	Silver Nanoclusters
AMPs	Antimicrobial Peptides
AMR	Antimicrobial Resistance
ARBs	Antibiotic Resistant Breakers
CA	Citric Acid
CAUITI	Catheter-Associated Urinary Tract Infection—Relevant Microbes
CCM	Curcumin
CD-MOF	Cyclodextrin-Based Metal–Organic Framework
CFR	Case Fatality Rate
CFs	Carboxymethylated Cellulose Fibers
CFU	Colony Forming Units
CNFS/CNFs	Cellulose Nanofibrils Films
COVID-19	Corona Virus Disease 19
CS	Chitosan
DNA	Deoxyribonucleic Acid
E330	Citric Acid
EC50	The 50% Effective Concentration
EC90	The 90% Effective Concentration
ECM	Extracellular Matrix
ESKAPE	*Enterococcus faecium*, *Staphylococcus aureus*, *Klebsiella pneumoniae*, *Acinetobacter baumannii*, *Pseudomonas aeruginosa*, And *Enterobacter* Spp.
EVD	Ebola Virus Disease
FDA	Food And Drug Administration
FT-IR	Fourier-Transform Infrared Spectroscopy
GA	Gallic Acid
Gm−	Gram-Negative Bacteria
Gm+	Gram-Positive Bacteria
GRGDS	(Gly-Arg-Gly-Asp-Ser) Peptide
GSH	Glutathione
H1N1	Influenza A Virus
HA	Hydroxyapatite
HAIs	Hospital Acquired Infections
HDP	Host Defense Peptides
HKUST-1	MOF, Hong Kong University of Science And Technology
HSV-2	Herpex Simplex Virus 2
HY	Hyaluronic Acid
ICP-AES/ICP-OES	Inductively Coupled Plasma Atomic Emission Spectroscopy/Inductively Coupled Plasma Optical Emission Spectrometry
LbL	Layer-by-Layer
MBC	Minimum Bactericidal Concentration
MERS-CoV	Middle East Respiratory Syndrome Coronavirus Infection
MIC	Minimum Inhibitory Concentration
MOF	Metal-Organic Framework
MRSA	Methicillin Resistant Streptococcus Aureus
NAM/NAMs	Nanoantimicrobial(s)
PCA	Polycaffeic Acid
PEG	Polyethylene Glycol
PEI	Polyethyleimine
PET	Polyethylene Terephtalate
PPA	Phenylpropanolamine
PVA	Polyvinyl Alcohol
PVP	Polyvinylpyrrolidone
RNA	Ribonucleic Acid
ROS	Reactive Oxygen Species
SARS-CoV-2	Severe Acute Respiratory Syndrome-Related Coronavirus 2
SDS	Sodium Dodecyl Sulphate
SEM	Scanning Electron Microscopy
TE	Green Tea Extract
TEM	Transmission Electron Microscopy
WHO	World Health Organization
